# Model Study of the Influence of Ambient Temperature and Installation Types on Surface Temperature Measurement by Using a Fiber Bragg Grating Sensor

**DOI:** 10.3390/s16070975

**Published:** 2016-07-01

**Authors:** Yi Liu, Jun Zhang

**Affiliations:** 1 School of Mechanical and Electronic Engineering, Wuhan University of Technology, Wuhan 430070, China; junzhang_918@126.com; 2 Digital Manufacture Key Lab of Hubei Province, Wuhan University of Technology, Wuhan 430070, China

**Keywords:** Fiber Bragg Gratings, fiber optics sensors, surface temperature measurement, error

## Abstract

Surface temperature is an important parameter in clinical diagnosis, equipment state control, and environmental monitoring fields. The Fiber Bragg Grating (FBG) temperature sensor possesses numerous significant advantages over conventional electrical sensors, thus it is an ideal choice to achieve high-accuracy surface temperature measurements. However, the effects of the ambient temperature and installation types on the measurement of surface temperature are often overlooked. A theoretical analysis is implemented and a thermal transfer model of a surface FBG sensor is established. The theoretical and simulated analysis shows that both substrate strain and the temperature difference between the fiber core and hot surface are the most important factors which affect measurement accuracy. A surface-type temperature standard setup is proposed to study the measurement error of the FBG temperature sensor. Experimental results show that there are two effects influencing measurement results. One is the “gradient effect”. This results in a positive linear error with increasing surface temperature. Another is the “substrate effect”. This results in a negative non-linear error with increasing surface temperature. The measurement error of the FBG sensor with single-ended fixation are determined by the gradient effect and is a linear error. It is not influenced by substrate expansion. Thus, it can be compensated easily. The measurement errors of the FBG sensor with double-ended fixation are determined by the two effects and the substrate effect is dominant. The measurement error change trend of the FBG sensor with fully-adhered fixation is similar to that with double-ended fixation. The adhesive layer can reduce the two effects and measurement error. The fully-adhered fixation has lower error, however, it is easily affected by substrate strain. Due to its linear error and strain-resistant characteristics, the single-ended fixation will play an important role in the FBG sensor encapsulation design field in the near future.

## 1. Introduction

Surface temperature is an important parameter in clinical diagnosis, equipment state control, and environmental and safety monitoring fields. It can reveal the hot and cold level of the measurement object, evaluate the physiological health situation [1] and running state of equipment [2], and diagnose diseases and device faults. High-accuracy surface temperature measurement technology is a key point to implement exact evaluation and diagnosis. The Fiber Bragg Grating (FBG) temperature sensor is a new temperature sensor and is utilized widely [3,4,5]. Compared to thermocouple or thermal resistor sensors, it possesses a fused silica wire as a signal route, which can effectively reduce thermal transmission of signal wires and improve the measurement accuracy [6]. In addition, the FBG sensor, characterized by high security, strong non-interference, small size, light weight, easy installation, and low costs, satisfies the harsh manufacturing environment [7]. It is an ideal choice to achieve high accurate measurement for equipment surface temperature. For traditional temperature sensors, many methods of placing temperature sensors on the surface of the measurement object, such as adhesive bonding, mounts installing, tape fixing, etc., are standard methods to obtain the surface temperature [8]. However, these methods still cannot avoid measurement error. Hennecke et al. [9] discussed the measurement error induced by a local heat sink on the surface. It is a measurement error induced by the high thermal conductivity of a thermocouple. Zvizdic [10] established a model of surface temperature measurement errors of thermocouples in vertical natural convection cooled channels. Kuznetsov et al. [11] discussed the influence of special glues and pastes on errors of temperature measurements by thermocouples. For the FBG temperature sensor, a new temperature sensor, the effect of the ambient temperature and installation types of sensors on precision of surface temperature measurement are often overlooked. Generally speaking, the cross-sensitivity of the FBG temperature sensor is only considered when it is fixed. Soumen et al. mounted a single FBG near a tool tip by using a tape to eradicate the cross-sensitivity issue [12]. The influence of conducting heat on the surface and installation on the measurement has not been discussed.

In order to fully reveal the relationship between surface temperature measurement errors and the installation types of FBG temperature sensors, we established a thermal transfer model of surface temperature measurement with the FBG sensor and propose a calibration system of surface temperature measurement error. The measurement errors which are induced by three installation types are theoretically analyzed and experimentally tested. According to the results of the theoretical and experimental analysis, two effects which determine measurement error are found.

## 2. Surface Temperature Measurement Principle of FBG Sensor

### 2.1. Principle of FBG Sensor

**Figure 1:**
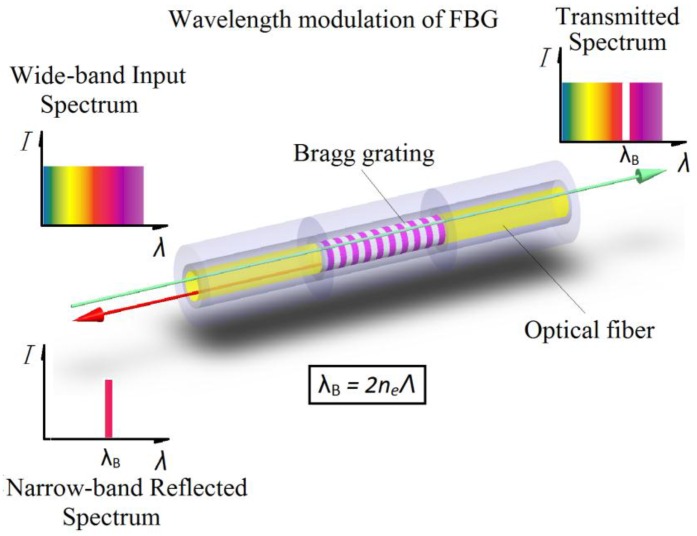
A schematic representation of a Bragg grating with incident, reflected, and transmitted light beams.

The FBG consists of a periodic modulation of the refraction along the fiber core, as shown in Figure 1. When a broadband light signal is injected into the optical fiber, most of the wavelength of light will reflect weakly on each of subsequent plate. At a particular wavelength which satisfies the Bragg interference condition, an intense reflected signal is generated. The particular wavelength is called the Bragg wavelength, *λ_B_*, which is determined by Equation (1).

(1) $$\lambda_{B}=2n_{eff}\cdot \Lambda$$

where *λ_B_*, the Bragg grating wavelength, is the free space center wavelength of the input light that will be back-reflected from the Bragg grating; Λ is the grating periodicity of the FBG; and *n_eff_* is the effective refractive index of the fiber core at the free space center wavelength. These two factors will be affected by changes in strain and temperature. For isotropic and homogeneous strain, the change of wavelength $\Delta \lambda_{B}$ is

(2) $$\Delta \lambda_{B}=\lambda_{B}(1-P_{e})\varepsilon_{z}+\lambda_{B}(\alpha_{\Lambda }-\alpha_{n})\Delta T$$

where $\varepsilon_{z}$ and ΔT are the change of strain and temperature in the FBG, and $P_{e}$ is an effective strain-optic constant. It is defined as:

(3) $$P_{e}=\frac{n_{eff}^{2}}{2}[p_{12}-v(p_{11}-p_{12})]$$

The parameters $p_{11}$ and $p_{12}$ are components of the strain-optic tensor, and v is Poisson’s ratio.

For Ge-doped silica-core fiber $p_{11}=0.113$, $p_{12}=0.252$, v=0.16, and $n_{eff}=1.482$, $P_{e}=0.3012$. In Equation (2), the parameter $\alpha_{\Lambda }=\frac{1}{\Lambda }\cdot \frac{\partial \Lambda }{\partial T}$ is the thermal expansion coefficient for the fiber (approximately 0.55 × 10^−6^ for silica). The parameter $\alpha_{n}=\frac{1}{n_{eff}}\cdot \frac{\partial n_{eff}}{\partial T}$ is the thermal-optic coefficient, which is approximately equal to 8.6 × 10^−6^ for Ge-doped silica-core fiber. Using these parameters and the Expression (2), the shift in the Bragg grating center wavelength due to strain and temperature changes is given by Equation (4):

(4) $$\Delta \lambda_{B}=1.048\text{ pm}/\mu \varepsilon \cdot \varepsilon_{z}+13.7\text{ pm}/{}^{\circ}C\cdot \Delta T$$

For a free FBG (floating on the specimen), only temperature can affect the center wavelength. Hence, this free FBG can be used to measure temperature. The wavelength shift of FBG temperature sensor is given by Equation (5):

(5) $$\Delta \lambda_{B}=13.7\text{ pm}/{}^{\circ}C\cdot \Delta T$$

For a FBG bonded on the specimen, it can sense both the changes of surrounding temperature and strain transferred from the specimen. The wavelength shift of the bonding sensor is given by Equation (4).

### 2.2. Temperature Field Model of FBG

**Figure 2:**
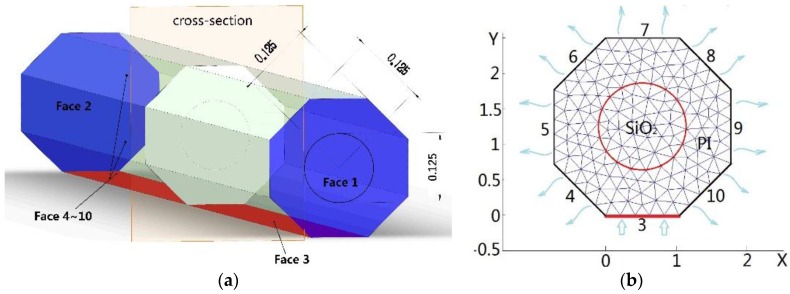
Temperature field model of FBG sensor. (**a**) 3D model of FBG sensor and (**b**) 2D heat conductivity model on the cross-section.

When a FBG is fixed on a hot surface, it is in contact with the surface in a narrow line and the remainder of cylindrical surface contacts with air. Heat energy transfers from the hot surface to the optical fiber and dissipates into the surrounding air by heat convection. In order to simplify the model, we suppose the optical fiber is a thin and long wire with an octagonal cross-section. The diameter of its inscribed circle is equal to the diameter *D* of the optical fiber 250 micrometer (including core, cladding, and coating). The installation length of the optical fiber is 20 mm. The heat transfer model of the FBG mounted on the surface is shown in Figure 2a. The bottom face, Face 3, is in contact with the heat source and its temperature kept constant at T_0_. The two end faces (Faces 1 and 2) of the optical fiber is so small that we consider them as heat insulation with the surroundings. The remaining side faces (Faces 4–10) are in contact with the air. The convection heat-transfer coefficient is *h* and the surrounding temperature is $T_{\infty }$. According to symmetry, each cross-section of the optical fiber has a uniform temperature distribution along the axis of the fiber. The three-dimensional temperature field can be simplified into a two-dimensional temperature field as shown in Figure 2b. The temperature field of the optical fiber is determined by the steady-state thermal conductivity Equation (6) without heat generation.

(6) $$\frac{\partial^{2}T}{\partial x^{2}}+\frac{\partial^{2}T}{\partial y^{2}}=0$$

On Face 3, the temperature is a constant T_0_; therefore, the Dirichlet boundary condition is applied as follows:

(7) $$T(x,0)=T_{0}$$

On Face *i* (*i* = 4, 5, …, 10), the Robin boundary condition [13] is given, as follows:

(8) $${K\vec{n_{i}}\cdot (\frac{\partial T}{\partial x}+\frac{\partial T}{\partial y})|}_{\Sigma =Face\_i}=h(T-T_{\infty })$$

where $\vec{n_{i}}$ is the normal unit vector of Face *i* and K is the conductivity of material.

A finite element method (FEM) is employed to resolve Equation (6) with boundary condition Equations (7) and (8). The material thermal properties and structure size for FBG sensor used in the numerical simulation are listed in Table 1.

**Figure 3:**
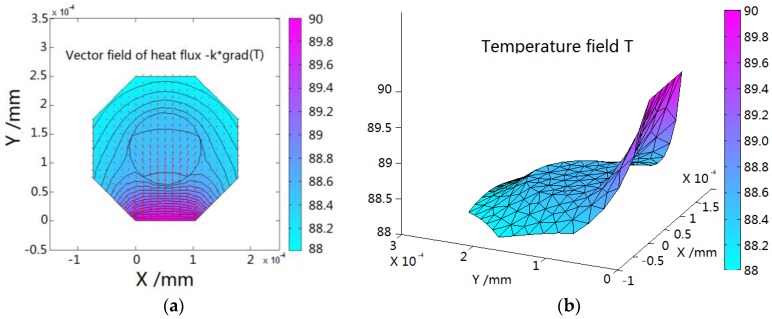
(**a**) Heat flux and (**b**) temperature field on optical fiber cross-section.

The simulation result of heat flux and temperature field on optical fiber cross-section is shown in Figure 3.

When the FBG is used to measure the temperature of the hot surface, only one side of the fiber contacts with the hot surface; therefore, the heat energy flows from this edge to the other side and dissipates into the surrounding air, as in Figure 3a. Along the heat flow direction, the temperature reduces. The rate of reduction (gradient of temperature) is determined by the thermal conductivity of the FBG material and convection condition. The thermal conductivity of the fiber core is greater than that of the coating layer. Thus, the temperature field on the cross-section of the fiber core which is used to detect temperature is even, but it is lower than the real temperature of the surface measured as shown in Figure 3b. The difference between the average temperature of the fiber core and real hot surface temperature is the measurement error.

### 2.3. Numerical Analysises of Measurement Error of FBG Sensors

**Figure 4:**
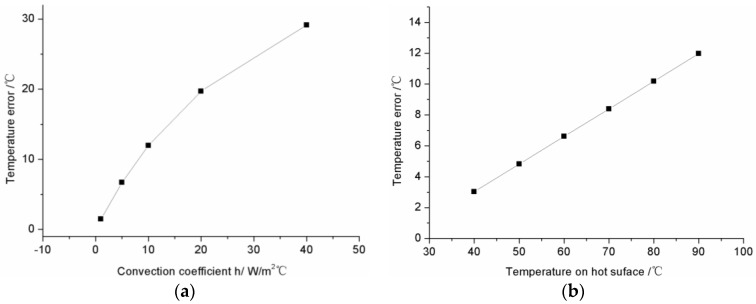
Measurement error numerical analysis of FBG Sensor. (**a**) The influence of convection on temperature error; (**b**) The influence of temperature difference between surrounding and hot surface on temperature error.

In the industrial environment, the thermal convection between the sensor and surroundings can influence measurement error. A variety of convection coefficients, *h* = 1, 5, 10, 20, 40 W/(m^2^·°C), are proposed to study the influence of the convection condition on measurement error of the FBG sensor. Other parameters are listed in Table 1. The simulation results of temperature error are shown in Figure 4a. As the convection coefficient increase, the temperature error increases rapidly. The reading of the sensor is lower than the real temperature on the hot surface, which is determined by the heat flow direction. The convection coefficient values in Figure 4a represent several typical measurement environments (such as *h* = 1 for a closed system in the laboratory, *h* = 5 for a small open system in the laboratory, *h* = 10 for an open system outdoors, *h* = 20 for fan convection in a workshop, and *h* = 30 for air-cooling in equipment). Under different work environments, FBG sensors show distinctly different error levels.

Under the same convection conditions, the measurement error is still affected by the temperature of hot surface measured. The temperature of the hot surface measured, $T_{0}$ = 40, 50, 60, 70, 80, 90 °C, is changed to study the dependent relationship between the measurement error of the FBG sensor and the real temperature of hot surface. In order to simulate the open system in the laboratory, a convection coefficient equal to 10 W/(m^2^·°C) is employed. Other parameters are listed in Table 1. The simulation results are shown in Figure 4b. As the measured temperature increases, the temperature error increases linearly. When 90 °C surface temperature is measured by the FBG sensor, the error induced by thermal transfer is 11.9 °C.

## 3. Measurement Errors of the FBG Sensor in Different Installation Types

### 3.1. Experimental Principle and Method

**Figure 5:**
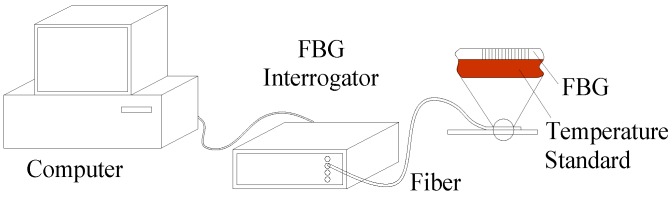
Surface error measurement principle schematic.

Measurement errors of a surface-mounted FBG temperature sensor are the difference between the result of the measurement and the true value of what is being measured. Generally speaking, the true value is difficult to obtain. To study the measurement error of the FBG temperature sensor on the surface, a surface-typed temperature standard must be fabricated. It is a reference whose temperature is known. A calibrated FBG temperature sensor is fixed on it with different installation types, as in Figure 5. The measurement errors are obtained by comparing the difference between the reading of the FBG sensor and the temperature of the surface-typed temperature standard.

### 3.2. Surface-Typed Temperature Standard Setup

**Figure 6:**
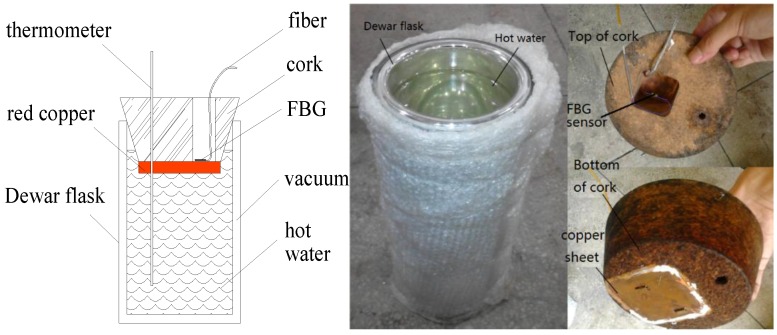
Principle schematic and experimental setup of a surface-typed temperature standard.

As shown in Figure 6, the experimental set-up for the temperature standard consists of the following devices: a Dewar flask, a cork, a piece of red copper which is fixed under the bottom of the cork, and a thermometer. The Dewar flask is filled with hot water. The thermometer is a standard mercury-in-glass type (Grade II) and verified in accordance with the verification regulation of standard mercury-in-glass (II). The thickness of the copper sheet is 1 mm and it is inundated with hot water. The temperature difference of the two sides of the copper sheet is very small (about 0.2 °C). The upside temperature of the copper sheet is approximately equal to that of the hot water. The change of the water temperature is very slow (about 0.3 °C/h) because of the big specific heat of water and the heat preservation property of the vacuum in the Dewar flask. Thus, the copper sheet is an ideal quasi-static-state surface-typed temperature standard. Its temperature is in accordance with that of the hot water in the Dewar flask. The thermometer is dipped into the water and used to measure the temperature accurately. Its reading is regarded as the temperature of the surface-typed temperature standard.

### 3.3. Experimental Sensor and Installation Types

**Figure 7:**
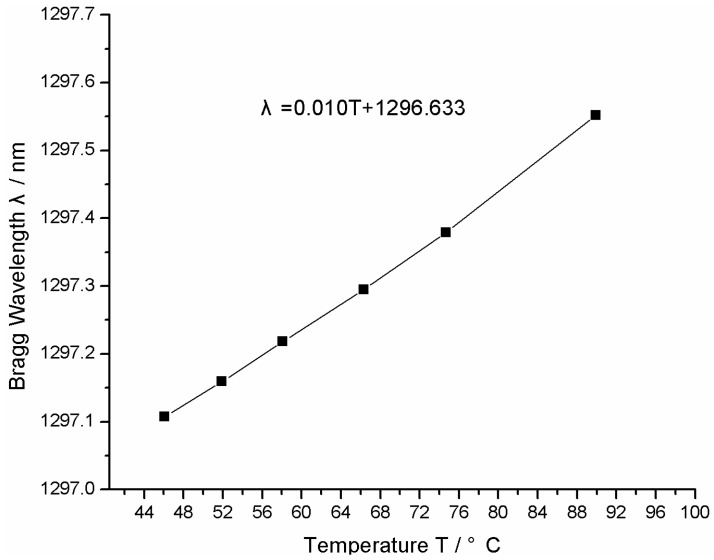
Temperature sensitivity curve for the FBG used in the experiment.

A FBG temperature sensor (its center wavelength is 1297 nm) is dipped into the hot water in the temperature standard setup. The temperature of hot water is changed six times from 46–92 °C and measured by the standard mercury-in-glass thermometers (Grade II). The reflected wavelength of the FBG is recorded by the FBG interrogator (MOI MS130). The calibrated curve is shown in Figure 7. The relationship between the temperature *T* and center wavelength *λ* of the FBG temperature sensor can be fitted as follows:

(9) λ=0.010T+1296.633

The temperature sensitivity of the FBG sensor is about 10 pm/°C.

**Figure 8:**
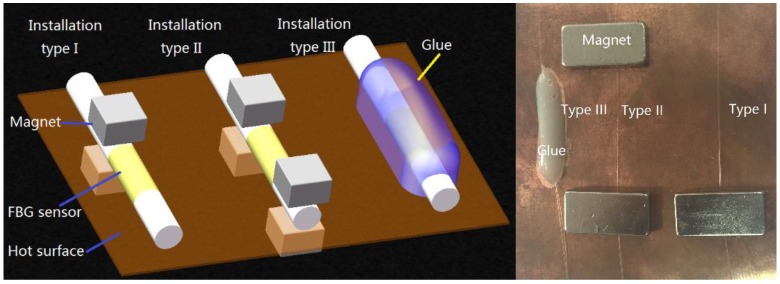
Schematic diagram and photograph of three common installation types of sensor.

General speaking, there are three installation types are employed in practice measurement. They are single-ended fixation, double-ended fixation, and full fixation. Three calibrated FBG temperature sensors are installed on the surface of the red copper sheet of the temperature standard setup with three different installation types. In Figure 8, the installation type I is the single-ended fixed model. The red copper sheet is the surface-typed temperature standard which simulates the surface of measured object. The FBG sensor is fixed on the red copper sheet by two magnets. One magnet is on the upper surface of the copper sheet and presses the fiber. Another magnet is under the copper sheet and can be pulled by the upper one. Since the FBG is only fixed at one end, it can freely stretch and cling to the red copper sheet. In installation type II, the FBG sensor is fixed on the red copper sheet by four magnets at two ends of the FBG. Installation type III is the adhesive bonding model. The FBG is pasted on the red copper surface by the acrylate adhesive and is fixed fully.

## 4. Results and Discussions

Fixation modes of sensors greatly influence surface temperature measurement results. Three fixation modes are discussed in this paper, which are the single-ended fixation with magnets, the double-ended fixation with magnets, and the full fixation with adhesive. Suppose the mercury thermometer reading is *T*_1_, the FBG sensor reading is *T*_2_, and the surface temperature measurement error is Δ*T*. Thus, Δ*T* = *T*_1_ − *T*_2_. The surface temperature measurement errors are tested under three different conditions.

### 4.1. Measurement Errors of Sensor under Three Different Types of Installation

**Figure 9:**
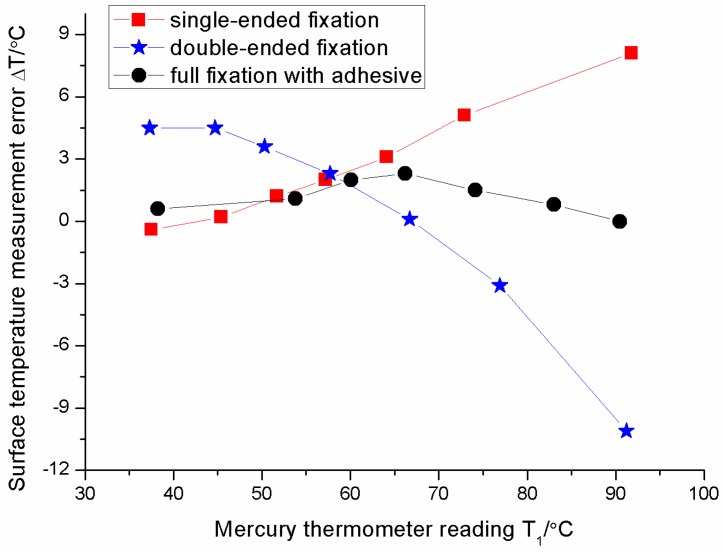
Surface temperature measurement errors of sensor under three different types of installation.

Ambient temperature is 23 °C in the experiments. The surface measurement errors, Δ*T*, of the FBG sensor under three different types of installation are shown in Figure 9. Three curves show the different change trends. The measurement errors of the sensor with the single-ended fixation increase, those with double-ended fixation decrease, and those with fully-adhered fixation fluctuate as the surface temperature rises. How are so prominent differences generated?

### 4.2. Influence of Installation Type of FBG on Measurement Error

**Figure 10:**
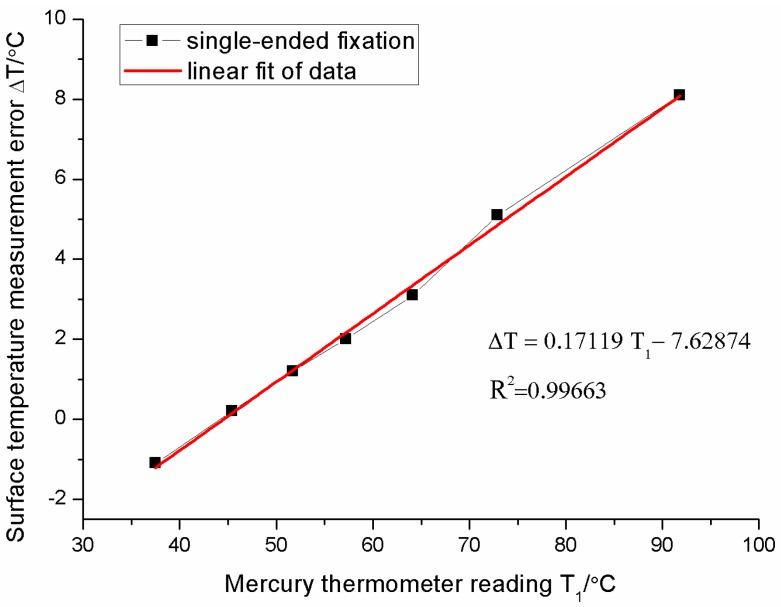
Surface temperature measurement errors of the sensor with the single-ended fixation.

For the single-ended fixation, Figure 10 shows the measurement error increases as the surface temperature increases. The relationship between errors and surface temperatures is approximately linearly dependent. The error is about 8 °C (percentage error is about 8/92 = 8.7%) when the surface temperature is 92 °C. The relationship between the error Δ*T* and the surface temperature *T*_1_ can be fitted as follows:

(10) $$\Delta T=0.17119T_{1}-7.62874$$

The difference between the real surface temperature and reading of FBG sensor is caused by the gradient of temperature on the surface. Each FBG sensor has a sensing point which is used to sense the temperature. The sensing point detects the temperature around itself, rather than the temperature of the object or surface. There is a serious temperature gradient between a hot surface and the cool ambience. Thus, the weak deviation between the sensing point and hot surface will induce significant measurement error. Moreover, the hotter the surface is, the greater the temperature gradient is and the more the measurement error will be. We call this phenomenon the “gradient effect”. The experimental results are in accord with simulation results in Figure 4b. Only the slopes of two lines are slightly different. It is caused by the different convection coefficients between the experiment and the simulation. Since the FBG can freely stretch on the surface of the measured object, it is impossible to be affected by the strain of the measured object. It is a highly robust installation method in the surface temperature measurement where there is an intense stress effect on the substrate. The remainder of the trouble is the large error when the installation method is used in the high-accuracy surface temperature measurement. Considering the stable linear error relationship, the compensation technology could resolve this problem satisfactorily. Therefore, the installation method, single-ended fixation, properly becomes a new research focus in the FBG sensor encapsulation design field.

**Figure 11:**
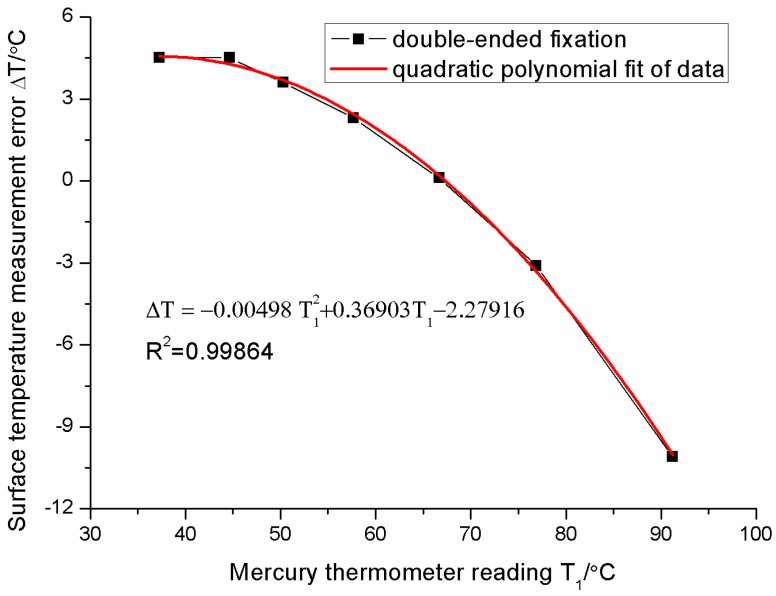
Surface temperature measurement errors of sensor with the double-ended fixation.

For the double-ended fixation, the measurement errors of the sensor change slowly, and then decrease rapidly as the surface temperature increases in Figure 11. The relationship between errors and surface temperatures is non-linearly dependent. The error at about 65 °C is zero. When the surface temperature exceeds 66 °C, the error Δ*T* becomes negative. This indicates that the readings of the FBG temperature sensor are higher than those of the surface. The largest error is about −11.1 °C (percentage error is about −12.2%) when the surface temperature is 91 °C. The relationship between the error Δ*T* and surface temperature *T*_1_ can be fitted as follows:

(11) $$\Delta T=-0.00498T_{1}^{2}+0.36903T_{1}-2.27916$$

This abnormal phenomenon may be due to two effects. One is the previous “gradient effect”. The bare FBG is also influenced by the gradient of temperature on the surface in the double-ended fixation. Another effect is caused by the substrate expansion. The FBG sensor is fixed at two ends of the Bragg grating. It cannot freely stretch. The coefficient of linear thermal expansion of the substrate (red copper) is much greater than that of fiber (SiO_2_). When the temperature rises, the FBG expands together with the expansion of substrate. The remarkable substrate-induced expansion of the FBG causes the increase of FBG sensor readings *T*_2_ and counteracts the decline of the readings induced by the temperature gradient effect. We call the phenomena as the “substrate effect” which is also determined by Equation (4). Thus, the thermal strain effect of the substrate will change the temperature gradient effect and disturb measurement results for the double-ended fixation. It is harmful to the measurement of surface temperature. In addition, the mechanical strain in the substrate also influences measurement results.

**Figure 12:**
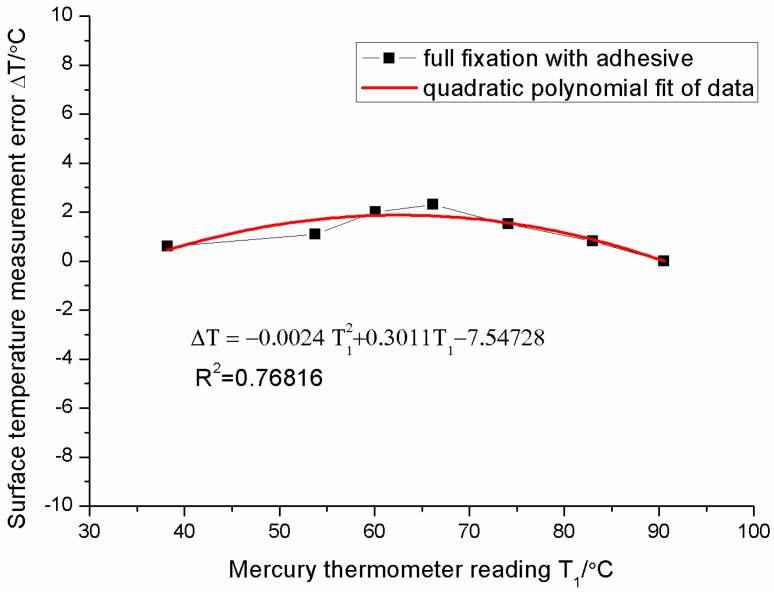
Surface temperature measurement errors of sensor with the full fixation.

For the fully-adhered fixation, the measurement error of the sensor increases, and then decreases as the surface temperature increases in Figure 12. The fluctuation of the measurement error is no more than 2 °C (percentage error is about 3%) in the temperature range of 35~95 °C. The relationship between the error Δ*T* and the surface temperature *T*_1_ can be fitted as follows:

(12) $$\Delta T=-0.0024T_{1}^{2}+0.3011T_{1}-7.54728$$

The measurement errors of the sensor with the full fixation are similar in the change trends to that with double-ended fixation. However, the measurement error of the sensor with the fully-adhered fixation is smaller than that with double-ended fixation. This may be due to the adhesive effect. The adhesive layer has two functions. Firstly, the adhesive layer increases the thickness of the conducting heat layer between the fiber core and the environment and thermal resistance, thus reducing the gradient of the temperature between the fiber core and the surface of the substrate. It works as a jacket on the fiber to prevent the thermal dissipation. The gradient effect is restrained and the measurement error reduces. Secondly, the thickness of the adhesive layer is about 2~3 mm and thicker than the substrate (1 mm red copper). The rigidity of the adhesive layer is close to that of the substrate. The thermal expansion of the substrate will be restrained by the adhesive layer on it. Therefore, the substrate effect is restrained and the measurement error is also reduced. Both of the effects bring about a great decline in the measurement error. Finally, it shows about 2 °C error in the range of 35–95 °C. The sensor with the fully-adhered fixation shows little error in this experiment, however, one must pay attention to the measurement condition in practice. The measurement error will change as the material and the dimension of substrate, and the stress in the substrate, change. Particularly, the stress-induced change of the Bragg wavelength is much larger than that induced by temperature in some machine parts. Therefore, the installation method, fully-adhered fixation, is only available in the measuring object whose temperature is close to the surroundings temperature and in which there is no stress.

## 5. Conclusions

The ambient temperature and installation types of FBG sensors are an easily overlooked issue. However, they greatly influence equipment surface temperature measurement results. High-accuracy surface temperature measurement is a challenging task. In order to reveal error sources, a theoretical analysis, a thermal transfer model of the FBG, and a calibration system of the surface temperature measurement error system are proposed. The measurement errors of the FBG temperature sensor with three different fixation types are tested. Experimental results show that two effects influence measurement results. The gradient effect results in an increasing linear error and the substrate effect results in a decreasing non-linear error with increasing surface temperature. The measurement error of the FBG sensor with single-ended fixation is determined by the gradient effect and is a linear error which is not affected by the substrate strain and is good for the compensation process. The measurement error of the FBG sensor with double-ended fixation is determined by the two effects and the substrate effect is dominant. Thus, the measured reading is higher than the real surface temperature. The measurement error of the FBG sensor with fully-adhered fixation is similar to that with double-ended fixation. However the adhesive layer can reduce the two effects and the measurement error in 35–95 °C is smallest, thus, it is used widely in surface temperature measurement. However, it is not available in the measurement whose measured object is affected by substrate strain. Sometimes the strain effect will exceed the change of the Bragg wavelength induced by temperature. Therefore, the single-ended fixation, which has the strain-resistant ability and the stable linear error, will play an important role in the FBG sensor encapsulation design field in the near future.

## Figures and Tables

**Table 1:** Parameters in the numerical simulation.

Parameters	Values
Conductivity of Fiber Core K_f_, W/(m·°C)	1.4 (SiO_2_)
Conductivity of coating layer K_c_, W/(m·°C)	2 (Polyimide)
Convection coefficient of Coating *h*, W/(m^2^·°C)	1 (laboratory condition)
Radii of coating r_p_ (μm)	125
Surrounding Temperature $T_{\infty }$, °C	23
Temperature on hot surface $T_{0}$, °C	90
Radii of optical fiber r_f_, μm	62.5
